# A study of the effects of dance/movement therapy on parenting stress and emotions in mothers of children with autism spectrum disorder

**DOI:** 10.3389/fpsyt.2025.1465677

**Published:** 2025-03-07

**Authors:** Xiang Yang, Xiaomei Zhan, Xiafang Li, Yuqing Wang, Ziwei Kuang

**Affiliations:** Autism Sports Intervention Centre, College of Physical Education, Jiangxi Normal University, Nan Chang, Jiangxi, China

**Keywords:** dance/movement therapy, mothers of children with autism spectrum disorder, parenting stress, depression, anxious

## Abstract

**Objective:**

Currently, many scholars are working to improve the core symptoms of children with autism spectrum disorder, while neglecting the mental health of caregivers of children with ASD. This study examined the effectiveness of dance/movement therapy (DMT) in reducing parenting stress in mothers of children with autism spectrum disorder and whether depression and anxiety mediated the effects thereof.

**Methods:**

Forty mothers of children with autism spectrum disorder were recruited in Nanchang, China, and divided into an experimental group (20) and a control group (20). The subjects were assessed before and after 12 weeks of dance/movement therapy (DMT) using the Parenting Stress Index/Short Form (PSI-SF), Self-Rating Depression Scale (SDS), and Self-Rating Anxiety Scale (SAS) as the assessment tools.

**Results:**

The results found that parenting stress, depression, and anxiety scores of mothers of children with autism spectrum disorder were significantly reduced after the dance/movement therapy (DMT) intervention.

**Conclusion:**

The mediating effects of depression and anxiety were significant, indicating that dance/movement therapy (DMT) is effective in reducing the levels of parenting stress, depression, and anxiety in mothers of children with autism spectrum disorder, and can indirectly play a role in reducing the levels of parenting stress in mothers of children with autism spectrum disorder by reducing their depression and anxiety.

## 1 Introduction

Autism spectrum disorder (ASD) is a neurodevelopmental disorder (1) that severely affects the patient’s language function, social behavior, sensory perception, etc., and its high prevalence rate of 2.27% has attracted extensive attention from all walks of life (2). As research on ASD increases, family lineage studies have found that ASD have a familial aggregation. Parents of children with ASD are vulnerable to mental disorder (3). The severity of ASD symptoms, children’s social impairments and problematic behaviors can cause varying degrees of parenting stress for parents of children with ASD (4).

**Figure 1:**
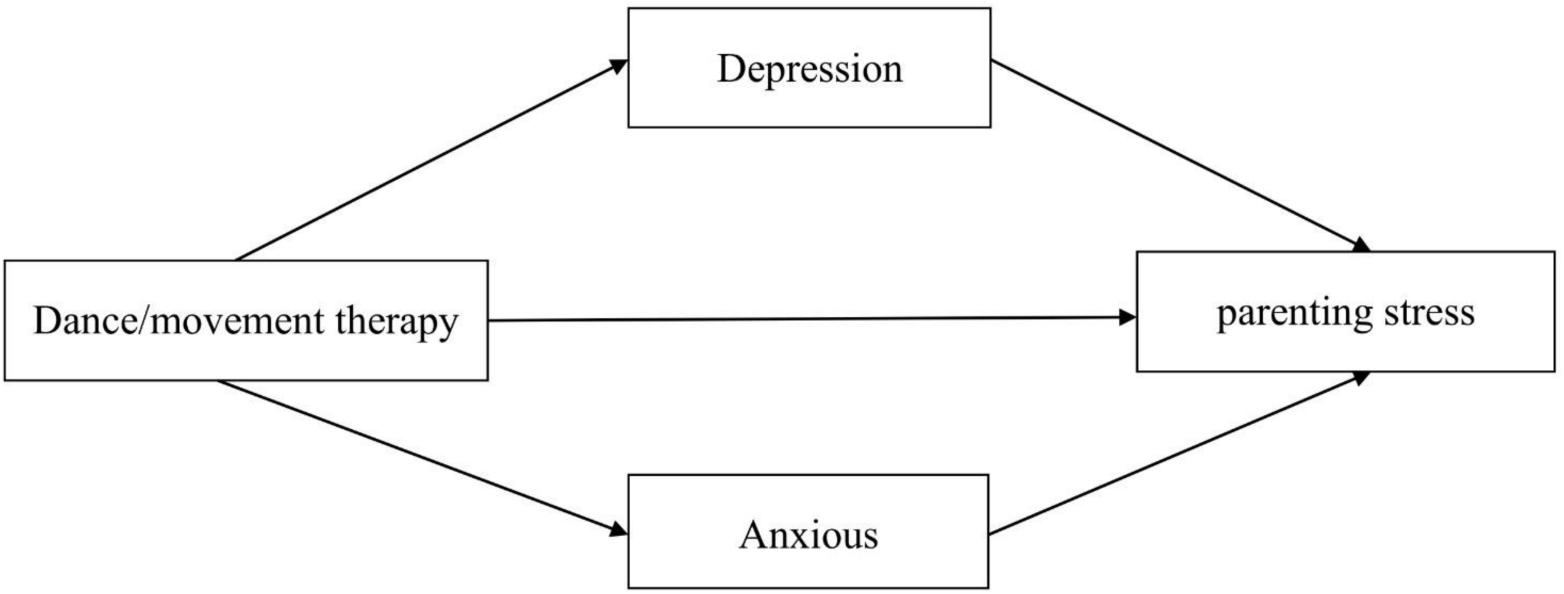
Model of the mediating hypothesis between depression and anxiety in the effect of DMT on parenting stress in mothers of children with ASD.

Parenting stress (PS) refers to the stress felt by parents or caregivers in the course of performing their parenting roles and parent-child interactions due to the influence of personality traits, parent-child interactions, children’s traits, and family situational factors (5). Parenting stress may lead to depression, anxiety, fatigue, increased neurological and hormonal activity (6). As mothers are usually the ones who take care of children with ASD in the traditional allocation of family roles in China, resulting in mothers often being troubled by parenting duties in the parent-child system, their parenting stress levels are much higher than fathers’ (7). Mothers of children with ASD also experience higher levels of parenting stress than mothers of typically developing children, and excessive parenting stress is detrimental to the physical and mental health of mothers of children with ASD (8)., and may even cause a chain reaction among family members, which is not conducive to the treatment and prognosis of children with ASD (9). Thus, there is an urgent need to find a way to effectively reduce parenting stress and negative emotions among mothers of children with ASD. Dance/movement therapy (DMT), as a kind of psychotherapeutic movement, is special in that it uses the body as the main therapeutic medium, and has the characteristics of improvisation and creativity that distinguish it from other somatic psychotherapies involving the body (10), language and non-language combination, through the synchronization, expression, rhythm, and integration of eight therapeutic factors (11) of DMT to heal the individual’s sorrow, thus achieving the goal of integrating the body and mind. Based on this, the research used DMT as an intervention to investigate the effects of DMT on parenting stress, depression, and anxiety in mothers of children with ASD, and proposed the following hypotheses: ①DMT significantly reduces parenting stress in mothers of children with ASD; ②DMT prominently reduces depression and anxiety in mothers of children with ASD; ③Depression and anxiety play a mediating role in the effect of DMT on parenting stress in mothers of children with ASD (mediating hypothesis model is shown in **Figure 1**).

## 2 Objects and methods

### 2.1 Participants

Based on previous studies (12), the sample size was calculated using G*Power 3.1.9.7 software, setting β= 0.1, Power =1 -β= 90%, and significance level two-sided α= 0.05. It was calculated that the number of subjects should not be less than 16. Assuming a 20% sample shedding rate, the final included sample size was calculated to be no less than 20 cases in total.

In this study, 40 mothers of children with ASD were recruited through the ASD Movement Intervention Research Center of Jiangxi Normal University and the Jiangxi Province Mentally Disabled Family and Friends Association in Nanchang, and were randomly divided into 20 in the experimental group and 20 in the control group. All subjects signed the informed consent form. Inclusion criteria: (i) mothers of children with ASD aged 6-12 years with a confirmed diagnosis issued by a hospital; (ii) mothers as primary caregivers; (iii) current residence in Nanchang, Jiangxi and able to participate in offline dance/movement therapy (DMT); (iv) voluntary participation and able to complete the scale assessment.

### 2.2 Assessment tools

Participants were assessed at baseline and post-intervention at the ASD Sports Intervention Research Center at Jiangxi Normal University using several scales as follows.

Parenting Stress Index/Short Form (PSI-SF) (13) was compiled by Abidin and revised by Wenxiang Ren in Chinese. The scale has three dimensions (12 questions in each dimension): Parenting Distress (PD), measuring the extent to which individuals experience stress related to their parenting role (e.g., “I often feel that I can’t handle things very well”); and Parent-Child Dysfunction Interaction (PCDI), measuring parents’ perceptions of the relationships and interactions with their children (e.g., “My child rarely does things for me that make me feel good”), and Difficult Child (DC), measuring parents’ perceptions of behavioral characteristics that make their child difficult or easy to manage (e.g., “My child seems to cry or fuss more often than most children “), with Cronbach’s alpha coefficients ranging from 0.80 to 0.91. The scale was scored on a Likert-type 5-point scale ranging from 1 point for strongly disagree to 5 points for strongly agree, with a total score higher than or equal to 90 indicating that the respondent’s stress response in the parent-child system was high (14).

Both the Self-Rating Depression Scale (SDS) (15) and Self-Rating Anxiety Scale (SAS) (16) were developed by Zung, and their reliability and validity were 0.94 and 0.91 respectively. Both scales have 20 items and are scored on a Likert-type 4-point scale ranging from 0 for no or little time to 3 for most or all of the time, with the higher the total standard score, the greater the depression or anxiety level. The standardized cut-off values for the depression and anxiety scales were 53 and 50, and the reliability and validity were 0.94 and 0.91 respectively.

### 2.3 Experimental steps and procedures

**Figure 2:**
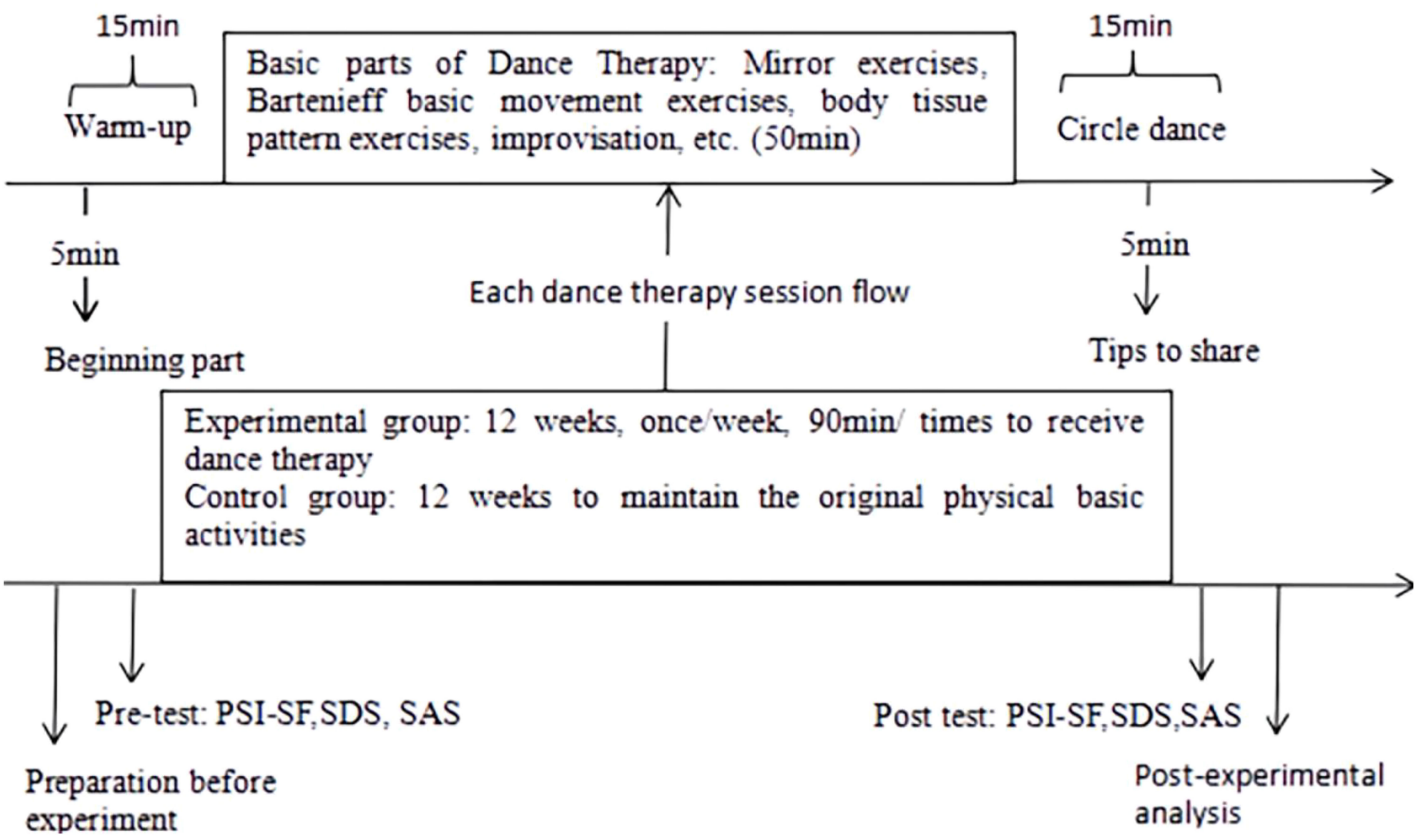
Flow chart of DMT experiment.

This study was a randomized controlled trial with three phases, and the flow of the DMT experiment is shown in **Figure 2**.

Pre-intervention preparation stage. With the help of the ASD Movement Intervention Research Center of Jiangxi Normal University and the Family and Friends Association of Mentally Disabled Persons in Jiangxi Province, the mothers of the recruited children with ASD were surveyed by visiting the actual situation, and intervenor investigated the subjects’ dance fundamentals and demographic characteristics to better develop the DMT program. Both the design and implementation of the program were led by the first author and another exercise prescriber; the first author had attended dance/movement therapy training at the Shanghai University of Sport (shown in **Table 1** for the specific DMT program). In accordance with the principle of voluntariness, after identifying the study participants, they were randomly grouped and pre-tested to assess the level of parenting stress, depression, and anxiety among mothers of children with ASD using PSI-SF, SDS, and SAS.

**Table 1:** 12-week DMT program objectives and basic activities.

Time	Activity Objectives	Specific Activity Setting
1	Leader and group members become acquainted with each other and build initial trust.	1. warm-up; 2. expressive name introductions; 3. breathing and vocalization exercises; 4. circle dance; 5. sharing of insights
2	Enhancing trust among members and restoring the natural developmental health patterns of the limbs.	1.warm-up; 2. Two-person Mirroring Exercises; 3. Bartineff Fundamentals; 4. circle dance; 5. sharing of insights
3	Increasing trust among members, understanding the movement traits one is good at or lacks, and exploring one’s own preferences and traits.	1.warm-up; 2. Two-person Mirroring Exercises; 3. Four kinds of Effort Element Experiences; 4. circle dance; 5. sharing of insights
4	Practicing how to express oneself internally while experiencing the feelings of others.	1.warm-up; 2. Group Mirroring Exercises; 3. ropes and relationships; 4. circle dance; 5. sharing of insights
5	Understanding movement states and emotional feelings during self-expression.	1.warm-up; 2.shape flow; 3.directional movement; 4. Bartenieff Fundamentals (BF), Basic 6; 5. circle dance; 6. sharing of insights
6-9	Exploring personal issues	1.warm-up; 2. Authentic Movement Exercises; 3. Two-step movement exercises; 4. circle dance; 5. sharing of insights
10	Experiencing bondage and freedom	1.warm-up; 2. movement, posture and positional experiences (using elastic bands as props); 3. circle dance; 4. sharing of insights
11	Experiencing social support systems	1.warm-up; 2. ropes and relationships; 3. group support (using elastic bands as props); 4. circle dance; 5. sharing of insights
12	Recalling groups	1.warm-up; 2. Group Mirroring Exercises; 3. sharing of self-experience and gains; 4. circle dance; 5. sharing of insights

DMT intervention phase. Mothers of children with ASD in the experimental group received DMT for 12 weeks, 1 session/week, 90 min/session (DMT program goals and basic activities are shown in **Table 1**). Each DMT intervention session consisted of a warm-up part, a basic part, and a relaxation part. The warm-up part mainly includes breathing exercises and power stretching exercises, which enable mothers of children with ASD to quickly relax, integrate into the dance group and enter the dance state. The basic part is centered around the intervention goals of each DMT intervention session, with different DMT intervention contents. For example, using pair or multi-person mirroring exercises, participants are asked to imitate each other’s movements and share their own experiences, and Bartenieff basic movement exercises (6 basic movements: breathing, core and limb ends, head and tail, upper and lower body, left and right half of the body, and the opposite side of the body) are used to improve the participants’ ability to express their body language and their awareness of their muscles and joints. Participants are encouraged to express themselves in the group through improvisational dance, sensing messages conveyed by group members with body contact, releasing emotions in dance, etc. The relaxation part was conducted in the form of a circle dance. Participants are asked to hold hands, make simple body movements around the circle and face the center of the circle, and share their dancing experience at the end of the circle dance. Mothers of children with ASD in the control group did not undergo any experimental treatment during this period and maintained their original basic body activities.

Post-intervention analysis phase: After the 12-week experiment, a post-test was conducted to assess the parenting stress, depression, and anxiety levels of mothers of children with ASD in both groups using the same format and scale as the pre-test. All the data obtained were also entered, the results of the assessment were summarized and sorted out, and the data were analyzed using statistical software and a thesis was written.

### 2.4 Controlling variables

In order to avoid the influence of the external environment on the experiment, the dance therapy was implemented in an exclusive dance room. To minimize information bias, participants were randomly grouped and the grouping was kept strictly confidential. Scale assessors were uniformly trained and did not participate in the intervention implementation. To reduce human subjective bias, the therapists strictly implemented the treatment protocol developed by the group to ensure the standardization of the experimental operation. To ensure the accuracy of the study results, the Harman one-way test was performed on the pre- and post-intervention data. The results showed that there were 13 and 12 common factors with eigenvalues greater than 1, and the percentage of variance explained by the first common factor was 21.97% and 26.30%, respectively, with the percentages being <40%, indicating that none of the data obtained from all the scales in this study had a serious common method bias.

### 2.5 Mathematical statistics

The Harman one-way test in SPSS 23.0 was used to test all data for common method bias. All data measured before and after the intervention were analyzed for normality using the K-S test to confirm that all data obeyed a normal distribution. Therefore, a paired sample t-test was used to analyze the effect of dance/movement therapy on parenting stress, depression, and anxiety in mothers of children with ASD. Independent samples *t*-test was used to compare the differences between the two groups of mothers of children with ASD in terms of parenting stress, depression, and anxiety. Model 4 in Process 3.3 was used to fit the depression in terms of the effect of DMT on parenting stress in mothers of children with ASD, anxiety mediating role model. The test levels were α=0.05. The effect size of the independent samples *t*-test and the paired samples *t*-test was expressed by Cohen’s d, taking the value of 0.2 to 0.5 as a small effect, 0.5 to 0.8 as a medium effect, and 0.8 or more as a large effect.

## 3 Results

### 3.1 Homogeneity test for experimental and control groups

20 mothers of children with ASD in each of the experimental and control groups. The average age of mothers of children with ASD in the experimental group was 41.42 ± 6.49 years old, and that of mothers of children with ASD in the control group was 39.83 ± 5.66 years old, the difference in age between the two groups was not significant (*t* = 0.90, *P* > 0.05). The average scores of the depression self-rating scale (SDS), anxiety self-rating scale (SAS), and parenting stress indicator short form (PSI-SF) of mothers of children with ASD in the experimental group were (54.75 ± 5.87), (44.60 ± 6.83) and (106.85 ± 14.72), respectively. While the average scores of SDS, SAS and PSI-SF of mothers of children with ASD in the control group were (54.85 ± 9.09), (47.00 ± 9.36), and (109.90 ± 15.69), respectively, with no statistically significant differences between the pretest scores of SDS, SAS, and PSI-SF of mothers of children with ASD in the two groups (*t*_SDS_=-0.04, *t*_SAS_=-0.92, and *t*_PSI-SF_=-0.63, all *P* values > 0.05), indicating that subjects in each group were from the same community.

### 3.2 Changes in parenting stress in the experimental group before and after DMT

As shown in **Table 2**, the total PSI-SF score as well as PD, PCDI, and DC scores of mothers of children with ASD in the experimental group are significantly reduced after 12 weeks of dance treatment (*t*_PSI-SF_ = 8.00, *t*_PD_ = 3.91, *t*_PCDI_ = 4.50, *t*_DC_ = 6.74; *P* < 0.01), Cohen’s d was 1.790, 0.875, 1.005, and 1.508, respectively, all of which were greater than 0.8, with larger effects. While no significant changes are observed in the control group of mothers of children with ASD when comparing pre- and post-tests (*P* > 0.05).

**Table 2:** Comparison of the PSI-SF dimension scores of mothers of children with ASD*(M ± SD)*.

Indicators	Control group (N=20)		Experimental group (N=20)	
	Pre-test	Post-test	Pre-test	Post-test
PSI-SF	109.90 ± 15.69	107.70 ± 13.90	106.85 ± 14.72	91.20 ± 9.45^**##^
PD	40.70 ± 8.71	41.45 ± 8.44	38.10 ± 6.64	34.70 ± 5.76^**##^
PCDI	33.70 ± 6.58	31.95 ± 7.60	32.65 ± 5.91	26.90 ± 3.64^**#^
DC	35.50 ± 6.03	34.05 ± 5.12	36.10 ± 8.36	29.60 ± 5.99^**#^

*Indicates intra-group comparison of pre- and post-test, ^#^indicates inter-group comparison of post-test; * or #indicates *P* < 0.05, ** or ##indicates *P* < 0.01.

**Figure 3:**
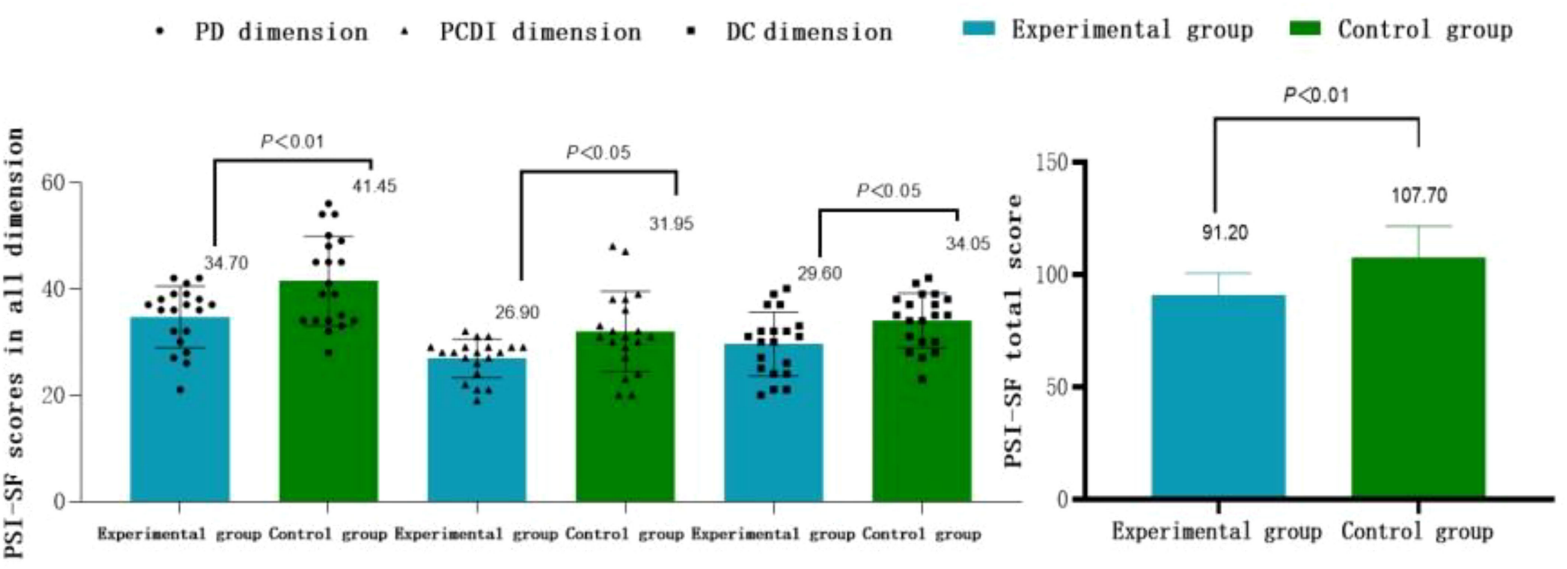
Comparison of the PSI-SF dimension scores and their total scores between the two groups of subjects after the intervention.

As shown in **Table 2** and **Figure 3**, the total PSI-SF score as well as the PD, PCDI and DC scores of mothers of children with ASD in the control group differ significantly from those of mothers of children with ASD in the experimental group after 12 weeks of DMT (*t*_PSI-SF_=4.38, *t*_PD_=2.95, *t*_PCDI_=2.68, t_DC_=2.52; *P*<0.05). Cohen’s d was 1.388, 0.934, 0.848, and 0.798, respectively, all greater than 0.8, with larger effects.

### 3.3 Changes in depression and anxiety in the experimental group before and after DMT

As shown in **Table 2**, the SDS and SAS scores of mothers of children with ASD in the experimental group significantly decreased after 12 weeks of DMT (*t*_SDS_=8.21, *t*_SAS_=10.15; *P*<0.01), Cohen’s d was 1.837 and 2.272, respectively, both greater than 0.8, with larger effects. While there is no remarkable change (*P*>0.05) in the control group of mothers of children with ASD when comparing pre- and post- tests.

**Figure 4:**
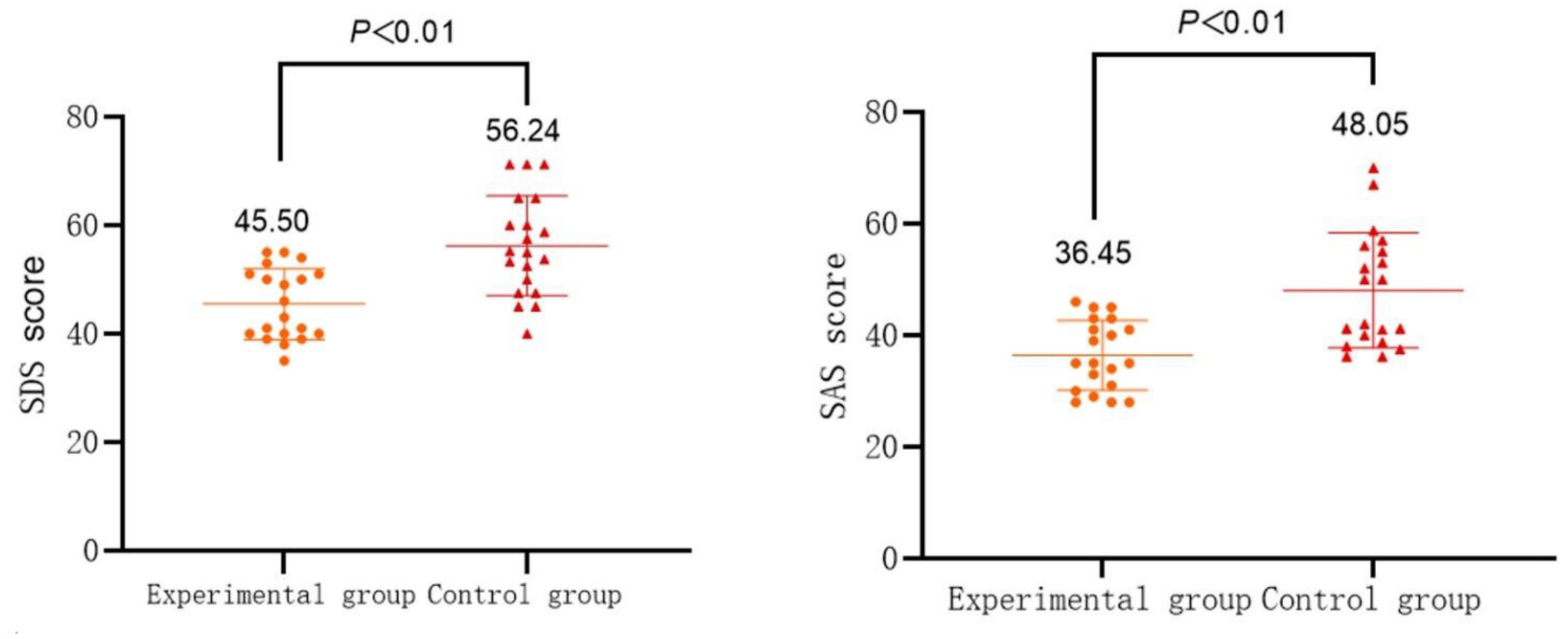
Comparison of SDS and SAS scores between the two groups of subjects after the intervention.

As shown in **Table 3** and **Figure 4**, after 12 weeks of DMT, both SDS and SAS scores of mothers of children with ASD in the control group differ dramatically from those of mothers of children with ASD in the experimental group (*t*_SDS_=4.26, *t*_SAS_=4.30; *P*<0.01). Cohen’s d was 1.347 and 1.360, respectively, both greater than 0.8, with larger effects.

**Table 3:** Comparison of SDS and SAS scores of mothers of children with ASD before and after the intervention (*M ± SD*).

Indicators	Control group (N=20)		Experimental group (N=20)	
	Pre-test	Post-test	Pre-test	Post-test
SDS	54.85 ± 9.09	56.24 ± 9.19	54.75 ± 5.87	45.50 ± 6.53^**##^
SAS	47.00 ± 9.36	48.05 ± 10.30	44.60 ± 6.83	36.45 ± 6.28^**##^

*Indicates intra-group comparison of pre- and post-test, ^#^indicates inter-group comparison of post-test; * or #indicates *P* < 0.05, ** or ##indicates *P* < 0.01.

### 3.4 A mediated model of depression and anxiety in DMT affecting parenting stress in mothers of children with ASD

With dance treatment as the independent variable (set “participating in DMT or not as a dummy variable: 1 for the experimental group and 0 for the control group), the PSI-SF total score after DMT as the dependent variable, and the SDS and SAS scores after DMT as mediating variables, Bootstrap test (standardized) for mediating effects of depression and anxiety is performed using Model4 in Process3.3.

As shown in **Table 4**, participating in DMT or not can negatively forecast parenting stress in mothers of children with ASD (*β*=-1.15, P<0.01), participating in DMT or not can directly predict depression in mothers of children with ASD (*β*=-1.12, *P*<0.01), and participating in DMT or not can directly predict anxiety in mothers of children with ASD (*β*=-1.13, *P*<0.01); when participating in DMT or not, depression, and anxiety can simultaneously predict parenting stress in mothers of children with ASD, the effect of participating in DMT or not to predict parenting stress in mothers of children with ASD is not remarkable (*β*=-0.04, *P*>0.05), and the effect of both depression and anxiety to predict parenting stress in mothers of children with ASD is significant, (*β*=0.63, *P*<0.01; *β*=0.35, *P*<0.01).

**Table 4:** Intermediary model tests.

Result Variables	Predictive variables	*R*	*R^2^*	*F*	*β*	*t*
Parenting	DMT	0.58	0.34	19.26^**^	-1.15	-4.39^**^
stress	DMT	0.57	0.32	18.14^**^	-1.12	-4.26^**^
Depression	DMT	0.57	0.33	18.50^**^	-1.13	-4.30^**^
Anxiety	DMT	0.98	0.96	326.61^**^	-0.04	-0.58
Parenting stress	Depression Anxiety				0.63	5.81^**^
					0.35	3.21^**^

*Indicates *P* < 0.05; **indicates *P* < 0.01.

**Figure 5:**
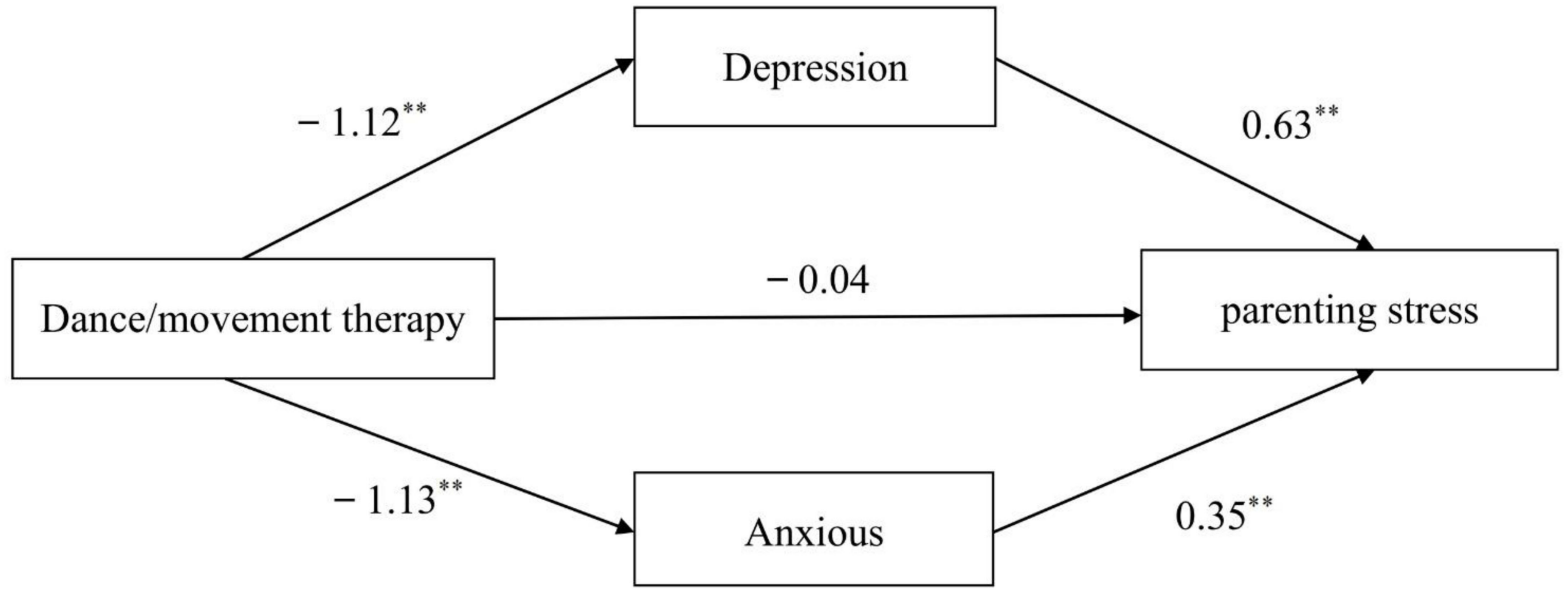
Fully mediated effect model of depression and anxiety in DMT affecting parenting. ** indicates *P* < 0.01.

As shown in **Table 5**, the results for the indirect effect of dance treatment through depression to parenting stress do not contain 0 (95% Boots CI=[-16.22,-4.57]), indicating a significant mediating effect of depression with an indirect effect of -10.19, which accounts for 61.76% of the total effect; the results for the indirect effect of dance treatment through anxiety to parenting stress do not contain 0 (95% Boots CI=[-11.15,-1.37]), indicating a significant mediating effect of anxiety with an indirect effect of -5.68, which represents 34.42% of the total effect; the results of the direct effect of dance treatment on parenting stress contain 0 (95% Boots CI=[0.56,-2.84]), indicating a non-significant direct effect. Thus, depression and anxiety play a fully mediating role in DMT affecting parenting stress. The mediating effect model is shown in **Figure 5**.

**Table 5:** Decomposition table of intermediary effects.

	Effect	BootSE	BootLLCI	BootULCI	Effect Ratio
Total utility	-16.50	3.76	-24.11	-8.89	
Direct effect	-0.63	1.09	0.56	-2.84	1.58%
Total indirect effect	-15.87	3.83	-23.44	-8.36	96.18%
Indirect effects of depression	-10.19	3.03	-16.22	-4.57	61.76%
Indirect effects of anxiety	-5.68	2.48	-11.15	-1.37	34.42%

## 4 Discussion

### 4.1 The effect of DMT on parenting stress in mothers of children with ASD

Parenting stress among mothers of children with ASD was significantly reduced in the experimental group after the DMT intervention. Aithal et al. (12) conducted DMT with mothers of children with ASD through multiplayer mirroring and movement exploration and found that DMT was effective in reducing the parenting stress of the subjects. Mirroring is a widely used technique in DMT and a powerful tool for cultivating empathy. Through mirroring movements, members of a DMT group can build bridges of physical dialog with each other and interact with each other through kinaesthetic empathy, breaking down inherent physical and psychological boundaries (10). The mirroring technique used in this study mainly included group mirroring and two-person mirroring. In DMT groups, participants establish a physical dialog with others through mutual imitation of body movements, response, and kinaesthetic empathy. This type of dialog can build bridges with each other, lead to interpersonal interactions, reduce an individual’s physical and psychological sense of self-preservation, and relieve stress. McGarry and Russo (17) also initially showed that when the mirror group imitates the action group’s performance due to feeling pleasure, its mirror neuron system and limbic system are activated at the same time, and the mirror group produces the same pleasurable feelings after modulation through the emotional action feedback system. Preliminary studies have shown that the movements, rhythms, and melodies in DMT also elicited positive effects at the physiological level in mothers of children with ASD, which could improve participants’ white matter integrity (18), increase the volume of gray matter in the parahippocampal gyrus (19), and enhance brain activation and brain network connectivity (20), which, in turn, promoted the physical and mental health of the participants by improving the individual’s cognitive functioning, motor perception, attentional control, and many other aspects.

### 4.2 The effects of DMT on depression and anxiety in mothers of children with ASD

This study showed that after 12 weeks of DMT, the levels of depression and anxiety of mothers of autistic children in the experimental group were reduced, indicating that DMT can significantly reduce the depression and anxiety of mothers of children with ASD. The results are similar to those of related studies, such as Plevin and Parteli (21) showed that DMT was effective in relieving anxiety and depression in patients with lung and breast cancer. Pylvänäinen et al. (22) conducted controlled experiments with depressed patients and indicated that DMT contributed to the recovery of depressed patients, a meta-analysis by Karkou et al. (23) also supports the positive effects of DMT on adult depression. In this study, exercises such as pair mirroring and circle dancing were used to enable participants to convey their inner feelings through expressive movements such as synchrony and echo, so that mothers of children with ASD could release their negative emotions in their movement expressions. At the same time, preliminary studies have shown that DMT activates the participant’s body and positively affects the physiological responses associated with depression and anxiety in mothers of children with ASD (24), which has the ability to strengthen the individual’s resistance to negative emotions (25). Additionally, Marian Chace, the founder of DMT, used group dancing as a group rhythmic activity, where rhythm serves as an externalizing medium whose infectious power organizes the actions of individual people. The group strength and sense of security created by mothers of children with ASD dancing together in the same musical setting can help mothers of children with ASD express their emotions more authentically. Moreover, the frequency of the musical rhythm itself resonates with the physiological rhythm of the child’s mother, generating stimuli that trigger the subcutaneous centers responsible for subjective emotions within the body of the child’s mother, and altering the activity of the autonomic nervous system, thereby regulating her negative emotions (26).

### 4.3 Mediating role of depression and anxiety between the effects of DMT on parenting stress in mothers of children with ASD

The results of this study suggest that DMT can reduce the level of parenting stress in mothers of children with ASD by improving their depression and anxiety. The process of mediating depression and anxiety can be divided into two stages: (1) depression and anxiety of mothers of children with ASD reduced after participating in DMT; (2) when depression and anxiety of mothers of children with ASD reduced, their levels of parenting stress also reduced. The results validate the hypothesis of this study and the view of related scholars that depression and anxiety are closely related to parenting stress. Yuh-Ming et al. (27) evaluated the parenting stress and depression status of 51 mothers of children with ASD and analyzed the relationship between the two. And it was found that the depressive status of mothers of children with ASD could predict their level of parenting stress. Neece et al. (28) also find that depression is highly correlated with parenting stress, while Hayes and Watson (6) suggests that depression and anxiety can be used as indicators to assess stress. On the one hand, dance as an aerobic exercise can make the human brain produce endorphins that can regulate the neuroendocrine system, making dancers feel happy and satisfied (29). During DMT mothers of children with ASD are stimulated by activities such as dance and improvisational movements to secrete more endorphins and experience more positive emotions, and this accumulation of positive emotions helps to alleviate negative emotions, which in turn produces an antidepressant effect, effectively repairing the psychological trauma that mothers of children with ASD experience as a result of their child having ASD, and parenting stress levels are thus reduced. On the other hand, dance as a form of expression allows participants to express emotions and feelings that are difficult to express in words, allowing the dancers to deescalate their negative emotions (22). DMT focuses on physical self-acceptance and expression, and mothers of children with ASD vent their negative emotions through abstract movements in dance, unlocking their self-imposed closures and enhancing their positive emotions (30), which consequently relieves the parenting stress of mothers of children with ASD. Thus, the mediating process by which DMT reduces the level of parenting stress in mothers of children with ASD by reducing their depression and anxiety can be explained as follows: the direct effect of DMT on parenting stress in mothers of children with ASD may not be significant, but depression and anxiety, as common negative emotions in individuals, are related to the level of stress in individuals, and the lower the level of depression and anxiety in individuals, the lower their level of parenting stress. Therefore, DMT may have an effect on parenting stress through the mediating effect of depression or anxiety in mothers of children with ASD.

### 4.4 Strengths and limitations

One of the strengths of the present study is that it is an intervention study with mothers of children with ASD as the target group; most of the current research has been on interventions for children with ASD, ignoring the psychological issues of their mothers. On the other hand, the present study is a controlled trial exploring the effects of DMT application, an area that has received less attention in the literature, and this study may inform how to improve the mental health of the parents of children with ASD.

This study also had several limitations. (1) The therapist assumed the dual role of researcher and may have had some bias. Throughout the DMT experiment, therapists avoided therapeutic interactions with participants as much as possible and strictly implemented the subject’s treatment protocol. However, future studies should implement blinding whenever possible to ensure the accuracy of the results. (2) This study used a no-intervention control group and only discussed the intervention effects of DMT on mothers of children with ASD; it did not compare DMT with other psychological interventions, but in the insight-sharing session, participants self-reported that DMT had a positive effect on mental health. Therefore, future research needs to focus on the differences in intervention effectiveness between DMT and other intervention methods to better analyze the impact of DMT on mothers of children with ASD. (3) The participants were all from the same region, and future research could go further in discussing the impact of DMT on mothers of children with ASD from different regions, cultural backgrounds, and socioeconomic statuses. (4) only two distress-related factors, “depression” and “anxiety,” were used as mediating variables to assess stress. More relevant factors (e.g., marital discord) could be included in the future to elucidate the potential pathways through which DMT acts on parenting stress in mothers of children with ASD. (5) It was assessed only at baseline and after the 1-week intervention, and future research could increase the number of assessments and follow-up measures to better explore trends in parenting stress among mothers of children with ASD under the influence of DMT.

## 5 Conclusion

DMT has a positive effect on the parenting stress, depression, and anxiety levels of mothers of children with ASD and can indirectly play a role in reducing the parenting stress levels of mothers of children with ASD by reducing their depression and anxiety. The effects of DMT on parenting stress in mothers of children with ASD could be explored in more depth in the future by designing different intervention groups, selecting participants from different regions and cultural backgrounds, and adding more mediating variables and assessments.

## Data Availability

The original contributions presented in the study are included in the article/supplementary material. Further inquiries can be directed to the corresponding author.
